# Psychodynamic group psychotherapy for family members of patients with psychoses

**DOI:** 10.1192/j.eurpsy.2025.2143

**Published:** 2025-08-26

**Authors:** M. Grah, B. Restek-Petrovic, V. Grosic

**Affiliations:** 1 University Psychiatric Hospital Sveti Ivan, Zagreb; 2 Faculty of Dental Medicine and Health, Osijek, Croatia

## Abstract

**Introduction:**

As part of our comprehensive early intervention program for psychotic disorders (RIPEPP), we have been conducting psychodynamic group psychotherapy for family members since 2005. This type of therapy intended for family members of patients that are motivated for psychological work and the correction of maladaptive forms of family interactions. The 90-minute sessions are held every two weeks under the guidance of a psychiatrist – group analyst.

At the beginning of this new experience, we applied group analytical principles in the group. Due to the large drop out and specific dynamics, we gradually changed the therapeutic technique and became more flexible and supportive.

During the years of therapy, with the strengthening of group cohesion, it was gradually possible to switch again from a more flexible and supportive method to the classic group analytical technique.

In this presentation, we will show vignettes from the therapeutic process.

**Objectives:**

An overview of the need to modify the group-analytic technique in the treatment of family members of individuals with psychotic disorders.

**Methods:**

Following the protocol of group sessions.

**Results:**

Over the course of the therapeutic process, the group members become more ready for group analytical work, which is adapted and applied in sessions in which they openly talk about themselves, their feelings and partner relationships. The modification of the group analytic technique is still at work with a continuously present psychodynamic understanding.

**Conclusions:**

A group-analytic approach is applied in the treatment of family members of individuals with psychotic disorders. The frequent occurrence of dropouts among group members highlighted the need to modify the group-analytic technique, with the potential to revert to the classical method by strengthening group cohesion and improving family dynamics.

**Disclosure of Interest:**

None Declared

